# Does obesity affect serum anti-müllerian hormone or inhibin B levels in polycystic ovary syndrome? Results of a systematic review and meta-analysis

**DOI:** 10.1186/s12958-026-01576-3

**Published:** 2026-06-15

**Authors:** Mei Jiang, Tao Huang, Daoyuan Jia, Ling Huang

**Affiliations:** 1 Beijing Research Institute of Chinese Medicine, Beijing University of Chinese Medicine, Beijing, China; 2 Department of Nephrology and Endocrinology, Dongzhimen Hospital, Beijing University of Chinese Medicine, Beijing, China; 3 Department of Traditional Chinese Medicine, Hudong Hospital, Shanghai, China; 4 School of Traditional Chinese Medicine, Beijing University of Chinese Medicine, No.11 Beisanhuandong Road, Chaoyang District, Beijing, 100029 P. R. China

**Keywords:** Polycystic ovary syndrome, Anti-müllerian hormone, Inhibin B, Obesity, Meta-analysis

## Abstract

**Background:**

Anti-Müllerian hormone (AMH) and inhibin B (INHB) are validated ovarian reserve biomarkers with key roles in polycystic ovary syndrome (PCOS) pathogenesis. Serum AMH has specific diagnostic value for PCOS, yet their differential expression and clinical significance in obese versus non-obese PCOS phenotypes remain unclear. To fill this gap, we performed the first systematic review and meta-analysis comparing serum AMH and INHB levels between obese and non-obese women with PCOS, and explored heterogeneity sources.

**Methods:**

Thirty-eight observational studies (*n* = 15,003) were systematically retrieved and quality-assessed via the Newcastle-Ottawa Scale (NOS), with all scoring ≥ 7. Meta-analyses calculated pooled standardized mean differences (SMDs) for AMH and INHB levels, with subgroup analyses stratified by geographic region, BMI category, and age. Meta-regression analyzed the effects of these factors on AMH levels; sensitivity analyses and publication bias assessments verified result robustness.

**Results:**

Obese PCOS women had significantly lower serum AMH (SMD: −0.16; 95% CI: −0.29 to −0.02; *P*=0.02; *I²*=86%) and INHB (SMD: −0.98; 95% CI: −1.60 to −0.36; *P*=0.002; *I²*=90%) than non-obese counterparts. Meta-regression found no significant associations between AMH levels and geography (*P*=0.6516), BMI (*P*=0.2598), or age (*P*=0.5353). Subgroup analyses showed the most pronounced AMH differences in BMI ≤28 kg/m² (*P*=0.01) and ≥18-year-old (*P*=0.02) subgroups, with reduced heterogeneity here. AMH results were stable in sensitivity analysis, while INHB findings relied on a single study; funnel plot asymmetry suggested potential publication bias for AMH studies.

**Conclusion:**

Obesity correlates with markedly reduced AMH and INHB in PCOS, indicating metabolic impacts on ovarian reserve biomarkers. These findings highlight the need for weight-stratified interpretation of such markers and further investigation into obesity-related mechanisms in PCOS.

**Supplementary Information:**

The online version contains supplementary material available at 10.1186/s12958-026-01576-3.

## Introduction

Polycystic ovary syndrome (PCOS), affecting 4%~21% of women worldwide, is a reproductive endocrine and metabolic disorder mainly characterized by (1) ovulatory dysfunction (infrequent or absent ovulation), (2) clinical and/or biochemical hyperandrogenism, and (3) polycystic ovarian morphology [1]. Clinically, it is mainly characterized by manifestations including menstrual disorders, infertility, hirsutism, and acne, with 60%~70% of patients being overweight or obese [1]. PCOS is often accompanied by insulin resistance and abnormal glycolipid metabolism, which play a crucial role in its pathogenesis. This combination of metabolic abnormalities may increase the risk of complications such as diabetes, cardiovascular diseases, endometrial lesions, and mental/psychological disorders, causing serious adverse effects on patients’ reproductive, physical, and mental health [2].

Anti-Müllerian hormone (AMH), also called Müllerian-inhibiting substance (MIS), is a glycoprotein member of the transforming growth factor-β (TGF-β) superfamily [3]. AMH is absent in primordial follicles but is expressed in early-stage primary follicles and secreted by granulosa cells of preantral and antral follicles [4–8], with expression persisting until follicles reach approximately 8 mm in diameter [9, 10]. Clinically, AMH serves as a biomarker for ovarian age, ovarian reserve, and the diagnosis and management of PCOS [11–18]. In PCOS, granulosa cell AMH production is 75 times higher than in those of healthy women [19], and plasma AMH levels are 2 ~ 3 times elevated than in controls [20, 21]. Women with AMH levels ≥ 4.45 ng/mL exhibit significantly higher PCOS risk than those with lower levels [22]. Evidence suggests that plasma AMH levels may quantify the severity of follicular dysfunction in PCOS [23, 24].

Inhibins are heterodimeric gonadal peptides that suppress follicle-stimulating hormone (FSH) secretion [25].Belonging to the transforming growth factor-β (TGF-β) superfamily, they consist of a common α-subunit linked by disulfide bonds to either a β_A_ (inhibin A, INHA) or β_B_ (inhibin B, INHB) subunit. While high-molecular-weight inhibin dimers exhibit biological activity in suppressing FSH, the free α-subunit is inactive [26]. INHB is primarily produced by the granulosa cells of ovarian primordial follicles and serves as a direct indicator of ovarian reserve function and follicular responsiveness [27]. Current studies report conflicting results on INHB levels in patients with PCOS. While some show no significant difference between patients with PCOS and controls [27], others demonstrate elevated INHB levels in PCOS [28, 29]. Interestingly, studies have shown that despite patients with PCOS having higher antral follicle counts (AFC), their serum INHB levels are significantly lower than those of healthy controls [30, 31]. Further study by Welt et al. [32] demonstrated significantly decreased INHB levels in the follicular fluid of patients with PCOS, suggesting a potential INHB production defect in PCOS.

Previous research indicates that obesity may directly impair AMH production at the granulosa cell level [33]. Although body mass index (BMI) serves as the standard clinical metric for obesity, studies report conflicting findings regarding its relationship with AMH levels: while some demonstrate an inverse correlation [34, 35], others show either positive [36, 37] or null associations [38–42]. Notably, PCOS populations consistently exhibit an inverse BMI-AMH relationship [43, 44], which has been independently replicated [43, 45]. In contrast, this association loses statistical significance in non-PCOS women [33, 46, 47]. Multiple studies have demonstrated a significant negative correlation between serum INHB levels and both BMI and fasting insulin levels in patients with PCOS [32, 48]. Welt et al. [32] further confirmed this relationship, showing that INHB and BMI were negatively correlated specifically in PCOS patients, but not in healthy controls. Interestingly, Pigny et al. [49] reported elevated INHB levels exclusively in non-obese PCOS patients (BMI ≤ 27 kg/m²), suggesting a BMI-dependent regulatory mechanism.

Whether obese women with PCOS have lower serum AMH or INHB levels compared to age-matched non-obese PCOS patients remains controversial, with current studies reporting inconsistent findings. To address this question, we conducted a systematic review and meta-analysis comparing erum AMH and INHB levels between obese and non-obese women with PCOS.

## Materials and methods

### Reporting guidelines

This systematic review and meta-analysis was conducted in accordance with the Preferred Reporting Items for Systematic Reviews and Meta-Analyses (PRISMA) guidelines and registered in the PROSPERO database (http://www.crd.york.ac.uk/PROSPERO, registration ID: CRD420251168313). The study also adhered to the Meta-analysis of Observational Studies in Epidemiology (MOOSE) checklist.

### Search strategy

The PICO (Population/Intervention/Comparison/Outcome) framework was defined as follows: P (women with PCOS), I (obese women with PCOS), C (age-matched non-obese women with PCOS), O (serum AMH or INHB levels). To identify eligible studies, a comprehensive literature search was conducted in the Cochrane Library, PubMed, EMBASE, and Web of Science databases up to September 2025, with no restrictions on language, geographic location, or journal type. The search strategy included the following keywords: “polycystic ovary syndrome” OR “PCOS” AND “Non-obese” OR “nonobese” OR “normal weight” OR “normal-weight” AND “Anti-Müllerian hormone” OR “AMH” OR “Müllerian inhibiting substance” OR “MIS” OR “Inhibins” OR “Inhibin-beta subunits” OR “Inhibin B” OR “Inhibin-beta” OR “Inhibin β” OR “INHB”.

### Inclusion and exclusion criteria

The inclusion criteria for eligible studies were as follows: (1) original studies involving human participants; (2) all study participants diagnosed with PCOS; (3) studies investigating the association between serum AMH or INHB levels and PCOS; and (4) studies reporting serum AMH or INHB levels in both obese women and age-matched non-obese women with PCOS.

Studies were excluded if they were conference abstracts or proceedings. For multiple publications reporting on the same study population, only the most recent publication or the one with the largest sample size was retained. Additionally, studies were excluded if participants had other endocrine disorders that could affect serum AMH/INHB levels or mimic PCOS, including diabetes mellitus, hyperprolactinemia, Cushing’s syndrome, congenital adrenal hyperplasia, androgen-secreting tumors, or active thyroid disease.

### Study selection and data extraction

**Fig. 1:**
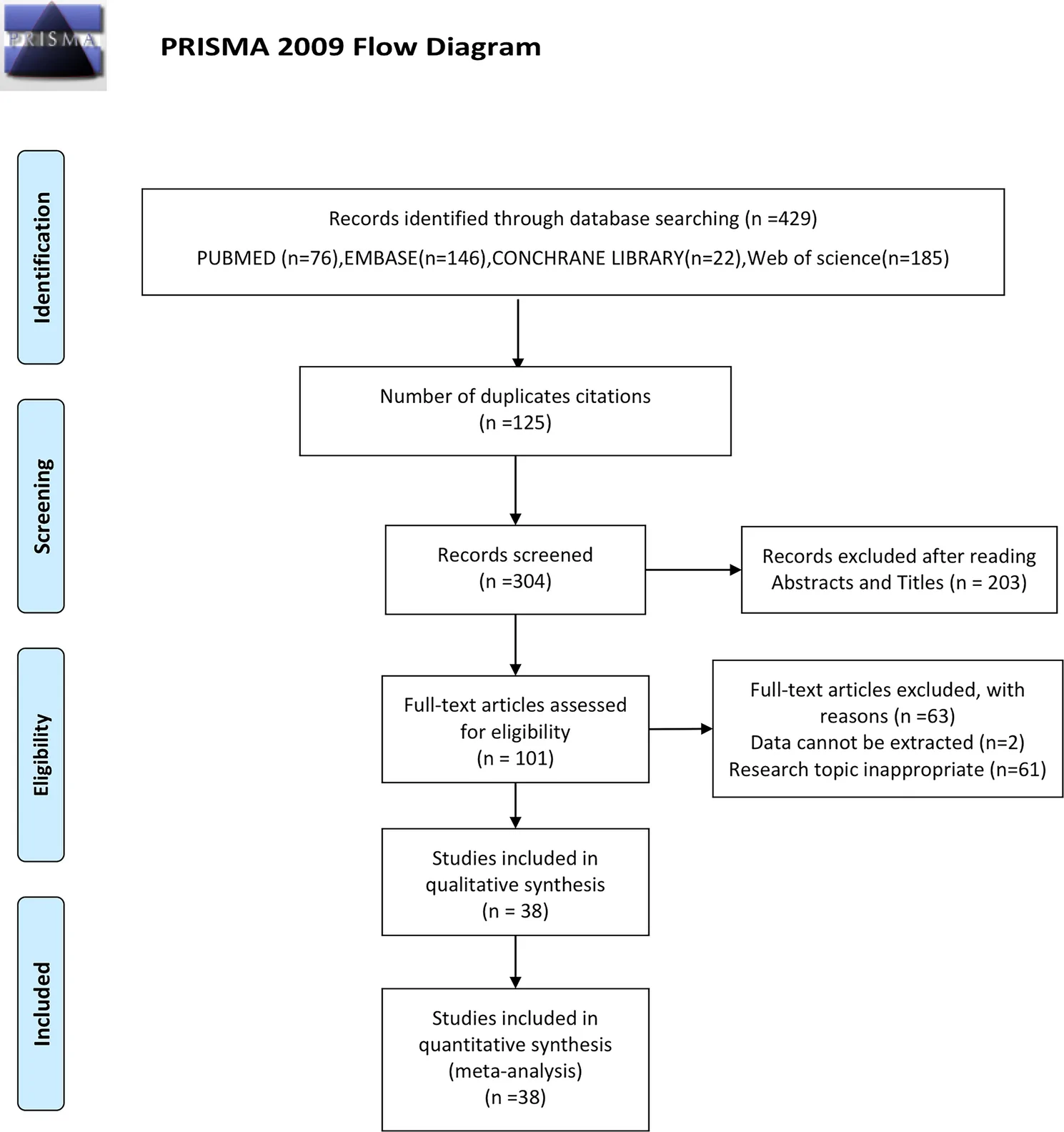
Flow chart of the selection process. From:Moher D, Liberati A, Tetzlaff J, Altman DG, The PRISMA Group (2009). Preferred Reporting Items for Systematic Reviews and Meta-Analyses: The PRISMA Statement. PLoS Med 6(7): e1000097. doi:10.1371/journal.pmed1000097

Two independent reviewers (MJ and TH) performed literature screening and data extraction, with discrepancies resolved by consensus or adjudication from a third reviewer (LH). The selection process followed three sequential steps: (1) removal of duplicates using EndNote; (2) screening of titles/abstracts against inclusion criteria; and (3) full-text assessment of potentially eligible studies. Figure 1 presents the PRISMA flow diagram of study selection. For eligible studies, serum levels of AMH and INHB (reported as mean ± SD) were extracted. When continuous variables were incompletely reported (e.g., missing SDs), corresponding authors were contacted to request raw data. For studies with unavailable responses, SDs were imputed from medians, interquartile ranges, or ranges using established methods [50, 51]. All extracted data underwent verification by DJ.

### Quality assessment

Two reviewers independently assessed the methodological quality of the included studies using the Newcastle-Ottawa Scale (NOS) system. The NOS evaluates three domains (selection, comparability, and exposure/outcome) with a maximum total score of 9; studies scoring ≥ 7 were considered to have high methodological quality [52].

### Statistical analysis

Meta-analyses were conducted on the extracted data using pooled standardized mean differences (SMDs) with 95% confidence intervals (CIs) to account for heterogeneity in AMH and INHB measurement units. The analyses evaluated serum AMH and INHB levels in PCOS patients. Heterogeneity was assessed using Cochran’s Q test (two-sided) [53], applying DerSimonian-Laird random-effects models when *I²* ≥ 50% and Mantel-Haenszel fixed-effect models otherwise [54, 55].

Subgroup and meta-regression analyses investigated associations between serum AMH/INHB levels and study characteristics to identify potential sources of heterogeneity. Sensitivity analyses were conducted by sequentially excluding individual studies to evaluate result stability. For meta-analyses including ≥ 10 studies, publication bias was assessed using funnel plots.

Meta-analyses were performed using RevMan software (version 5.3), while meta-regression analysis was conducted using R software (version 4.3.2), with statistical significance set at *P* < 0.05.

## Results

### Literature search

After screening titles and abstracts of 304 initially identified articles, 203 articles that did not meet the inclusion criteria were excluded. Of the remaining 101 articles subjected to full-text assessment, 63 were excluded (2 due to non-extractable data and 61 as irrelevant to the research topic), yielding 38 eligible observational studies for final inclusion (Fig. 1). These studies provided individual data from 7,803 obese women and 7,200 non-obese women [45, 48–90]. Table 1 summarizes the baseline characteristics of the included studies, detailing author information, publication year, geographical location, PCOS diagnostic criteria, sample size, age range, BMI values, measurement methods, and key findings.

**Table 1:** Summary characteristics of studies and participants

No.	Study	Region	PCOS diagnosis criteria	Sample size (*n*)		Age(y)			BMI(kg/m^2^)		Measuring method of AMH/INHB	Primary conclusion
				obese	non-obese	obese	non-obese		obese	non-obese		
1.	Pigny (2000) [49]	France (Lille)	NIH Criteria	21	20	25 ± 5.7	26 ± 4.7		31 ± 2.3	22 ± 2.5	Two-site ELISA (Serotec, Oxford, UK)	Serum INHB decreased in obese PCOS
2.	Cortet-Rudelli (2002) [48]	France (Lille)	NIH Criteria	71	63	27.4 ± 4.7			> 25	≤ 25	Two-site ELISA (Serotec, Oxford, UK)	Serum INHB decreased in obese PCOS
3.	Siow (2005) [56]	Canada (Ontario)	Rotterdam Criteria	19	12	14.9 ± 1.8	15.2 ± 1.8		33.8 ± 6.3	20.8 ± 2.6	ELISA	Serum AMH remained unchanged in obese PCOS
4.	Tsigkou (2008) [57]	Brazil (Belo Horizonte)	Rotterdam Criteria	93	52	18 ~ 42			26 ~ 51	19 ~ 25	ELISA(Diagnostic System Laboratories/Oxford Bio Innovation, Oxford, United Kingdom)	Serum INHB decreased in obese PCOS
5.	Pinhas-Hamiel (2009) [58]	Israel (Tel Aviv)	Rotterdam Criteria	14	16	15.2 ± 1.2	15.5 ± 1.7		29.6 ± 13.2	20.1 ± 2	ELISA (Diagnostic Systems Laboratory, Webster, TX, USA)	Serum AMH remained unchanged in obese PCOS
6.	Piouka (2009) [59]	Greece (Thessaloniki)	Rotterdam Criteria	100	100	25.35 ± 5.67	25 ± 5.28		30.75 ± 3.81	22.18 ± 1.75	ELISA (kit, DSL Laboratories, Webster, TX)	Serum AMH decreased in obese PCOS
7.	Skałba (2011) [60]	Poland (Katowice)	Rotterdam Criteria	33	54	26.0 ± 4.7	24.1 ± 3.5		29.0 ± 3.4	21.1 ± 2.2	ELISA (Immunotech a.s., Prague, Czech Republic)	Serum AMH remained unchanged in obese PCOS
8.	Hwang (2013) [61]	Korea (Seoul)	Rotterdam Criteria	34	141	26.1 ± 6.7	25.8 ± 5.5		> 25	≤ 25	ELISA (Immunotech version, Beckman Coulter, Marseille, France)	Serum AMH decreased in obese PCOS
9.	Cengiz (2014) [62]	Turkey (Istanbul)	Rotterdam Criteria	29	29	17.8 ± 1.74	18.2 ± 1.85		28.3 ± 3.46	20.1 ± 2.61	ELISA (Eastbiopharm, Hangzou, China)	Serum AMH remained unchanged in obese PCOS
10.	Aghadavod (2015) [63]	Iran (Kashan)	Rotterdam Criteria	20	19	29 ± 4.9	28.16 ± 4.1		28.4 ± 2.7	23 ± 1.9	ELISA (Beckman Coulter Immunotech, Villepinte, France)	Serum AMH decreased in obese PCOS
11.	Yang (2015) [64]	China (Taiwan)	Rotterdam Criteria	50	106	25.35 ± 8.4	24 ± 6.01		31.86 ± 4.38	21.6 ± 2.78	second-generation ELISA (Immunotech A Bechman Coulter Company, Marseilles, France)	Serum AMH decreased in obese PCOS
12.	Chiofalo (2017) [65]	Italy (Siena)	Rotterdam Criteria	29	24	30 ± 6	27 ± 4		44 ± 7	23 ± 3	ELISA (Beckman Coulter)	Serum AMH decreased in obese PCOS
13.	Ersoy (2017) [66]	Turkey (Istanbul)	Rotterdam Criteria	34	49	25.0 ± 3.2	25.5 ± 5.1		33.1 ± 2.3	22.5 ± 2.0	ELISA (Ansh Labs Webster, TX)	Serum AMH remained unchanged in obese PCOS
14.	Garin (2017) [67]	USA (PA)	NIH Criteria	45	20	31.29 ± 7.66	28.64 ± 7.18		39.3 ± 6.43	25.65 ± 3.75	Gen II ELISA (Beckman Colter, Brea, CA, USA)	Serum AMH decreased in obese PCOS
15.	Lefebvre (2017) [45]	France (Lille)	Rotterdam Criteria	30	30	18 ~ 36			> 25	≤ 25	ELISA (Beckman Coulter Immunotech, Villepinte, France)	Serum AMH decreased in obese PCOS
16.	Zhong (2017) [68]	China (Guangdong)	Rotterdam Criteria	54	94	29.00 ± 4.15		28.44 ± 3.97	≥ 24	< 24	ELISA	Serum AMH remained unchanged and serum INHB decreased in obese PCOS
17.	Xiong (2018) [69]	China (Shanghai)	Rotterdam Criteria	19	44	24.1 ± 3.2		23.4 ± 2.4	> 23	18 ~ 23	ELISA	Serum AMH remained unchanged in obese PCOS
18.	Kurek Eken (2019) [70]	Turkey (Aydın)	Rotterdam Criteria	39	40	28.44 ± 5.05		26.78 ± 5.19	≥ 30	18.5 ~ 24.9	ELISA (Ansh Labs Webster, TX)	Serum AMH increased in obese PCOS
19.	Yetim Şahin (2019) [71]	Turkey (İstanbul)	Rotterdam Criteria	29	23	16.64 ± 1.48 14.5 ~ 20		16.84 ± 1.36 14.5 ~ 20	1 ~ 2 SDS or SDS > 2	SDS < 1	ELISA (Beckman Coulter Company)	Serum AMH remained unchanged and serum INHB decreased in obese PCOS
20.	Liang (2020) [72]	China (Nanning)	Rotterdam Criteria	8	10	27.1 ± 3.5		25.7 ± 3.5	29.5 ± 4.8	20.7 ± 1.9	ECLIA (Beckman Coulter, Inc.)	Serum AMH increased in obese PCOS
21.	Paulson (2020) [73]	Sweden (Stockholm)	Rotterdam Criteria	10	10	18 ~ 40			37.4 ± 5.4	21.9 ± 1.8	ECLIA	Serum AMH decreased in obese PCOS
22.	Peigné (2020) [74]	France (Lille)	Rotterdam Criteria	42	29	28.1 ± 14.5		24.18 ± 13.05	35.76 ± 9.94	21.21 ± 6.87	ECLIA (Beckman Coulter)	Serum AMH remained unchanged in obese PCOS
23.	Simkova (2020) [75]	Czech Republic (Prague)	Rotterdam Criteria	10	9	29.5 ± 5.8		28.9 ± 7.4	35 ± 2.7	21.4 ± 3.2	ECLIA	Serum AMH remained unchanged in obese PCOS
24.	Luo (2021) [76]	China (Shenyang)	Rotterdam Criteria	106	50	30.72 ± 3.10		29.48 ± 2.94	27.13 ± 2.77	20.94 ± 1.55	ECLIA (Beckman Coulter)	Serum AMH decreased in obese PCOS
25.	Shi (2021) [77]	China (Shenyang)	Rotterdam Criteria	79	40	≥ 18			30.55 ± 4.03	21.99 ± 3.59	ECLIA	Serum AMH remained unchanged in obese PCOS
26.	Zhang (2021) [78]	China (Beijing)	Rotterdam Criteria	159	92	32.46 ± 3.16		30. 52 ± 3. 28	29.33 ± 3.21	21. 06 ± 1. 35	ELISA	Serum AMH decreased in obese PCOS
27.	Carmina (2022) [79]	Italy (Palermo)	Rotterdam Criteria	72	131	24.2 ± 5			35.04 ± 4.29	21.72 ± 2.01	ELISA (Gen II assay, Beckman Coulter, Brea, CA)	Serum AMH remained unchanged in obese PCOS
28.	Niepsuj (2022) [80]	Poland (Wroclaw)	Rotterdam Criteria	8	30	25.5 ± 4.47		27.07 ± 7.01	31 ± 1.61	22.21 ± 1.95	ELISA (Immunotech a.s., Prague, Czech Republic)	Serum AMH remained unchanged in obese PCOS
29.	Zeng (2022) [81]	China (Fujian)	Rotterdam Criteria	139	40	27.3 ± 5.02		28.3 ± 4.0	≥ 24	< 24	ECLIA (Roche Diagnostics GmbH)	Serum AMH decreased in obese PCOS
30.	Çatal (2023) [82]	Turkey (İstanbul)	Rotterdam Criteria	90	90	26.68 ± 4.55		25.54 ± 4.74	36.05 ± 3.89	26.58 ± 1.8	ECLIA (Siemens Healthineers Germany Advia Centaur XPT immunoassay analyzer )	Serum AMH remained unchanged in obese PCOS
31.	Gao (2023) [83]	China (Yunnan)	Rotterdam Criteria	40	38	25.26 ± 5.70		24.52 ± 4.70	31.40 ± 5.40	19.92 ± 3.51	NA	Serum AMH remained unchanged in obese PCOS
32.	Hynes (2023) [84]	North Carolina (Durham)	International PCOS Guideline	5156	4134	31.57 ± 3.72		31.3 ± 3.5	≥ 25	18.5 ~ 24.9	NA	Serum AMH remained unchanged in obese PCOS
33.	Zhao (2023) [85]	China (Liaoning)	Rotterdam Criteria	127	93	28.50 ± 4.36		27.61 ± 4.15	≥ 25	< 25	ELISA	Serum AMH decreased in obese PCOS
34.	Li (2024) [86]	China (Hubei)	Rotterdam Criteria	110	145	25.06 ± 4.77		24.22 ± 4.61	≥ 25	18.5 ~ 24.9	ECLIA	Serum AMH increased in obese PCOS
35.	Niepsuj (2024) [87]	Poland (Wroclaw)	Rotterdam Criteria	27	33	26.03 ± 3.21		25.15 ± 4.28	32.28 ± 4.95	21.06 ± 1.93	ELISA (Immunotech a.s., Prague, Czech Republic)	Serum AMH remained unchanged in obese PCOS
36.	Zhao (2024) [88]	China (Zhejiang)	Rotterdam Criteria	195	871	29 ± 2.99		29.18 ± 3.21	26.54 ± 1.49	21.65 ± 2.17	NA	Serum AMH decreased in obese PCOS
37.	Vale-Fernandes (2025) [89]	Portuga (Porto)	Rotterdam Criteria	37	31	32.16 ± 4.05		32.26 ± 3.83	31.67 ± 5.03	21.92 ± 1.95	ELISA (Beckman Coulter Access AMH, Immunotech, France)	Serum AMH remained unchanged in obese PCOS
38.	Wang (2025) [90]	China (Henan)	Rotterdam Criteria	625	388	28.6 ± 3.84		28.6 ± 3.2	27.86 ± 3.02	21.6 ± 1.5	NA	Serum AMH decreased in obese PCOS

*BMI* body mass index, *AMH* anti-Müllerian hormone, *INHB* Inhibin B, *PCOS* polycystic ovary syndrome, *ELISA* Enzyme-Linked Immunosorbent Assay, *NIH* National Institute of Child Health and Human Development, *ECLIA* enzyme chemiluminescence immunoassay, *NA* Not available

### Quality assessment

The quality assessment of the literature was performed using the NOS system, and all selected studies had a score of ≥ 7 (Table 2).

**Table 2:** The results of NOS star system scoring

No.	Study	Adequacy of Case Definition	Representativeness of Cases	Selection of Controls	Definition of Controls	Comparability of Cases and Controls	Ascertainment of Exposure	Same Method of Ascertainment for Cases and Controls	Non-Response Rate	Total
1.	Pigny (2000) [49]	1	0	1	1	1	1	1	1	7
2.	Cortet-Rudelli (2002) [48]	1	1	1	1	1	1	1	0	7
3.	Siow (2005) [56]	1	1	1	1	2	1	1	1	9
4.	Tsigkou (2008) [57]	1	1	1	1	2	1	1	0	8
5.	Pinhas-Hamiel (2009) [58]	1	1	1	1	2	1	1	1	9
6.	Piouka (2009) [59]	1	1	1	1	2	1	1	1	9
7.	Skałba (2011) [60]	1	1	1	1	2	1	1	1	9
8.	Hwang [61] (2013)	1	1	1	1	1	1	1	0	7
9.	Cengiz (2014) [62]	1	1	1	1	2	1	1	1	9
10.	Aghadavod (2015) [63]	1	1	1	1	2	1	1	1	9
11.	Yang (2015) [64]	1	1	1	1	2	1	1	1	9
12.	Chiofalo (2017) [65]	1	0	1	1	2	1	1	1	8
13.	Ersoy (2017) [66]	1	1	1	1	2	1	1	1	9
14.	Garin (2017) [67]	1	1	1	1	2	1	1	1	9
15.	Lefebvre (2017) [45]	1	1	1	1	2	1	1	0	8
16.	Zhong (2017) [68]	1	1	1	0	2	1	1	1	8
17.	Xiong (2018) [69]	1	1	1	1	2	1	1	1	9
18.	Kurek Eken (2019) [70]	1	1	1	1	2	1	1	1	9
19.	Yetim Şahin (2019) [71]	1	1	1	1	1	1	1	1	8
20.	Liang (2020) [72]	1	1	1	1	2	1	1	1	9
21.	Paulson (2020) [73]	1	1	1	1	2	1	1	0	8
22.	Peigné (2020) [74]	1	1	1	0	2	1	1	1	8
23.	Simkova (2020) [75]	1	1	0	1	2	1	1	1	8
24.	Luo (2021) [76]	1	0	0	1	2	1	1	1	7
25.	Shi (2021) [77]	1	1	1	1	2	1	1	1	9
26.	Zhang (2021) [78]	1	1	1	1	1	1	1	1	8
27.	Carmina (2022) [79]	1	1	0	0	2	1	1	1	7
28.	Niepsuj (2022) [80]	1	1	1	1	2	1	1	1	9
29.	Zeng (2022) [81]	1	0	1	1	2	1	1	1	8
30.	Çatal (2023) [82]	1	1	1	1	2	1	1	1	9
31.	Gao (2023) [83]	1	1	0	1	2	1	1	1	8
32.	Hynes (2023) [84]	0	1	1	1	2	1	1	1	8
33.	Zhao (2023) [85]	1	1	1	1	2	1	1	1	9
34.	Li (2024) [86]	1	1	1	1	2	1	1	0	8
35.	Niepsuj (2024) [87]	1	1	1	1	2	1	1	1	9
36.	Zhao (2024) [88]	1	1	1	1	2	1	1	1	9
37.	Vale-Fernandes (2025) [89]	1	1	1	1	2	1	1	1	9
38.	Wang (2025) [90]	1	1	1	1	1	1	1	1	8

### Meta-analysis results

**Fig. 2:**
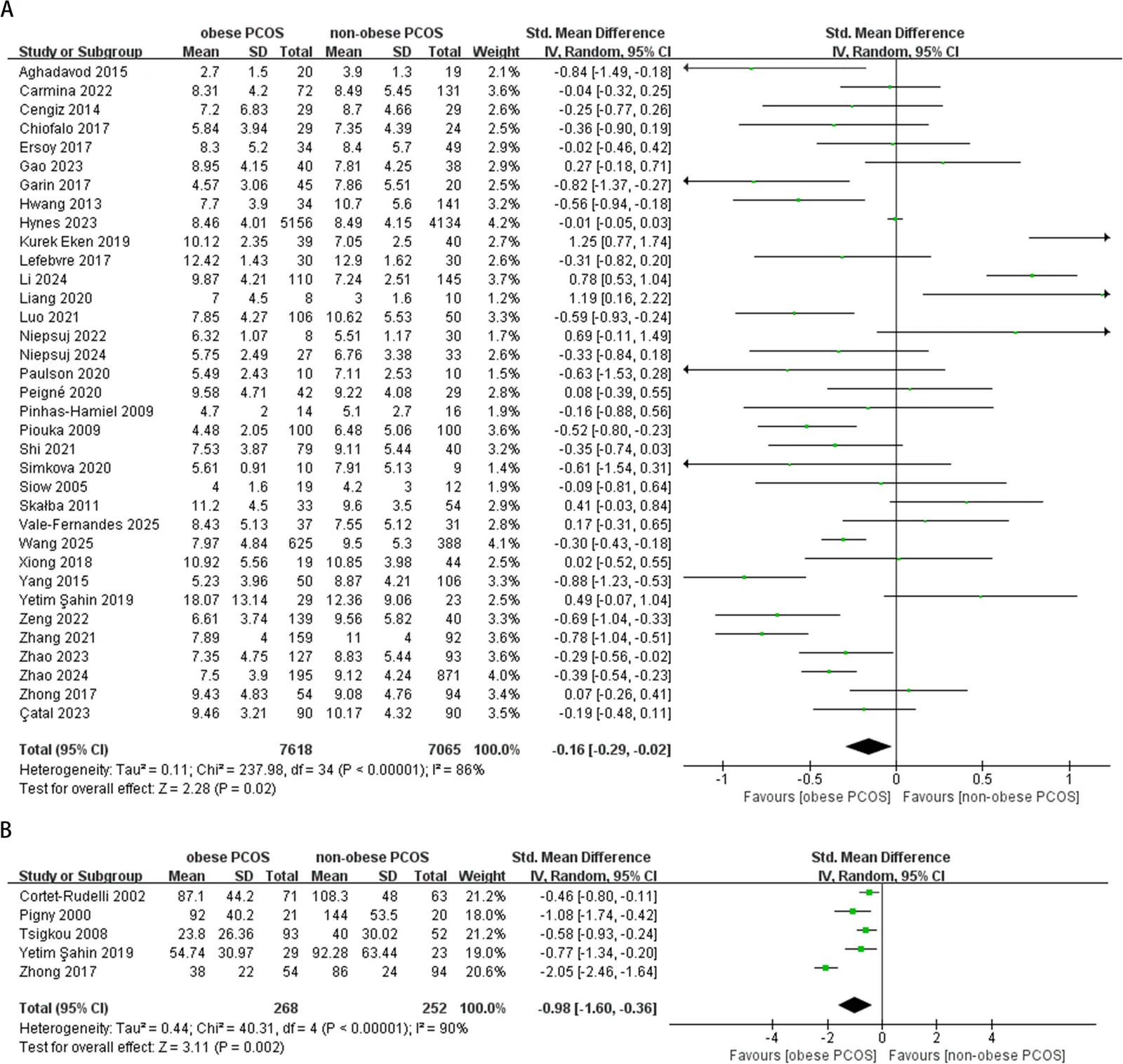
Forest plot of the levels of serum AMH and INHB levels in obese versus and non-obese women with PCOS. Weights are from random effects analysis. (**A**) Meta-analysis of serum AMH levels. (**B**) Meta-analysis of serum INHB levels. CI, confidence interval; SD, standard difference

Among thirty-eight studies (*n* = 14683) comparing AMH levels between obese and non-obese women with PCOS (Fig. 2A), significant inter-study heterogeneity was observed (*I²*=86%, *P* < 0.00001). The pooled analysis revealed that obese women with PCOS had significantly lower AMH levels than their non-obese counterparts (SMD: −0.16; 95% CI: −0.29 to − 0.02; *P* = 0.02).

Five studies (*n* = 520) compared serum INHB levels between obese and non-obese women with PCOS (Fig. 2B). Significant inter-study heterogeneity was observed (*I²*=90%, *P* < 0.00001). The pooled analysis indicated that obese women with PCOS had significantly lower serum INHB levels than their non-obese counterparts (SMD: −0.98; 95% CI: −1.60 to − 0.36; *P* = 0.002).

### Results of subgroup analysis

Substantial heterogeneity (*I²* > 50%) was observed across studies in this meta-analysis of serum AMH levels in women with PCOS. To explore potential sources of heterogeneity and improve the accuracy of comparisons between obese and non-obese women with PCOS, predefined subgroup analyses were performed, stratified by geographic variations, BMI stratification, and age.

Subgroup analysis by geographical region (Table 3) showed no significant differences in serum AMH levels between obese and non-obese women with PCOS in European (*P* = 0.31), Asian (*P* = 0.16), or North American populations (*P* = 0.31). Notably, heterogeneity was reduced in the European subgroup (*I²*=52%).

**Table 3:** Effect estimate and heterogeneity of subgroup analysis for serum AMH levels

Subgroup	Trials (*n*)	Sample size (*n*)	Effect Estimate SMD (95% CI)	*I* ^*2*^	*P*
Geographical location					
Europe	15	1252	−0.09 (−0.30, 0.08)	52%	0.31
Asia	17	4045	−0.18 (−0.42, 0.07)	90%	0.16
North America	3	9386	−0.27 (−0.80, 0.25)	76%	0.31
BMI(kg/m^2^) categories of obese women					
≤ 28	6	781	−0.36 (−0.63,−0.08)	65%	0.01
> 28	29	13,902	−0.11 (−0.26, 0.04)	87%	0.14
Age (years)					
≥ 18	31	14,512	−0.17 (−0.31,−0.03)	87%	0.02
< 18	4	171	0.01 (−0.35, 0.37)	27%	0.95

*CI* confidence interval, *SMD* standard mean difference, *BMI* body mass index

When stratified by the mean BMI of the obese group using the cutoff of 28 kg/m² (Table 3), obese women with PCOS in the ≤ 28 kg/m² subgroup had significantly lower serum AMH levels compared with their non-obese counterparts (SMD: −0.36; 95% CI: −0.63 to − 0.08; *P* = 0.01; *I²*=65%). In contrast, no significant difference was observed in the > 28 kg/m² subgroup (SMD: −0.11; 95% CI: −0.26 to 0.04; *P* = 0.14; *I²*=87%).

Age-stratified analyses (Table 3) demonstrated that obese women with PCOS aged ≥ 18 years had significantly lower serum AMH levels than their non-obese counterparts (*P* = 0.02), whereas no significant difference was observed in those aged < 18 years (*P* = 0.95). Notably, heterogeneity was substantially lower in the younger age subgroup (*I²*=27%).

### Meta-regression analysis

The geographical location, BMI, and age were used as the independent variables in the meta-regression analyses for AMH. Results (Table 4) indicated that no significant association between AMH levels and geographical location (*P* = 0.6516), BMI (*P* = 0.2598), or age (*P* = 0.5353).

**Table 4:** Meta-regression analysis to investigate the relationship between geographical location, BMI, age and AMH (*P* value)

Indicator	Geographical location	BMI	Age
AMH	0.6516	0.2895	0.5353

*AMH* Anti-Müllerian hormone, *BMI* body mass index

### Sensitivity analysis

Sensitivity analysis was conducted by sequentially excluding individual studies to evaluate result robustness. For serum AMH levels, the pooled effect size remained consistent after each exclusion, demonstrating robust meta-analysis outcomes [91]. In contrast, serum INHB analysis showed limited stability compared to the findings of Zhong et al. [68]. Notably, exclusion of the latter study completely resolved heterogeneity (*I²*=1%).

### Publication bias

**Fig. 3:**
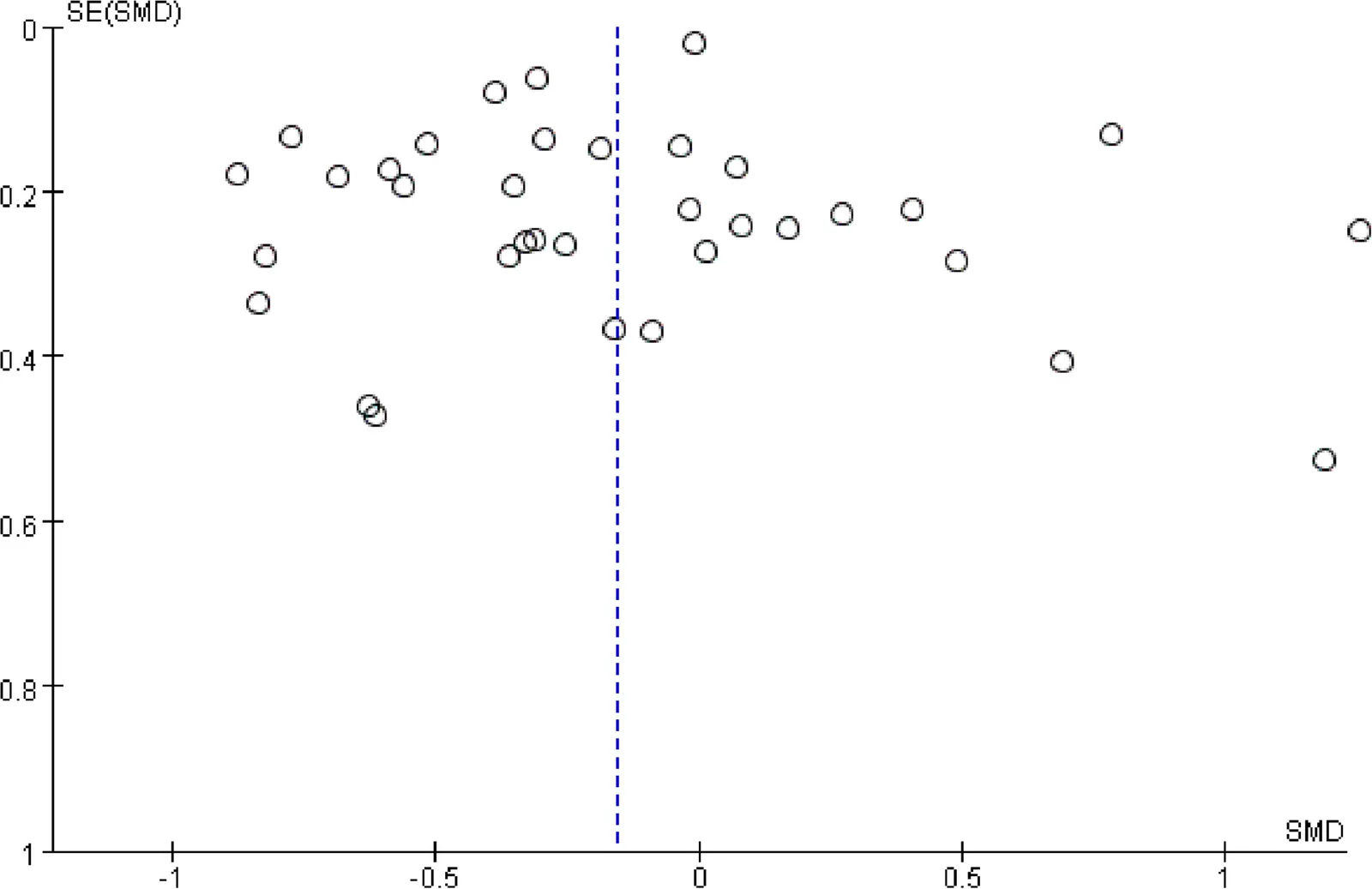
Funnel plot analysis of serum AMH levels

Systematic reviews may introduce publication bias when conclusions are drawn without incorporating unpublished evidence. In this analysis, funnel plot asymmetry suggested potential publication bias for serum AMH levels (Fig. 3). However, we did not assess publication bias for serum INHB levels, as the Cochrane Handbook for Systematic Reviews of Interventions (www.cochranehandbook.org) recommends that meta-analyses containing fewer than 10 studies have insufficient statistical power for reliable bias detection.

## Discussion

AMH and INHB are well-established biomarkers of ovarian reserve and PCOS pathogenesis [23, 27], with serum AMH demonstrating particular diagnostic value for PCOS [59, 92]. Nevertheless, their differential expression patterns in obese versus non-obese PCOS phenotypes remain poorly understood. To resolve this uncertainty, we performed the first comprehensive systematic review and meta-analysis of 38 studies (*n* = 15,003) comparing these biomarkers across BMI categories. Our stratified analyses by geography, BMI classification, and age revealed consistently lower serum AMH and INHB levels in obese PCOS patients compared to their non-obese counterparts. These findings indicate that while both biomarkers maintain their potential for PCOS assessment, their diagnostic performance in obese populations requires additional validation due to obesity-related metabolic influences. Consequently, clinical interpretation of ovarian reserve markers in PCOS should incorporate metabolic status as a key modifying factor.

It is noteworthy that, despite the significantly lower serum AMH levels in obese PCOS women compared to non-obese PCOS women, AMH levels in obese PCOS patients may still be markedly elevated relative to normal-weight healthy women without PCOS, as documented in previous studies [19, 20, 92]. This observation suggests that obesity attenuates—but does not reverse—the PCOS-associated AMH excess. Consequently, while the diagnostic utility of AMH for PCOS is preserved in obese populations, BMI-specific reference thresholds may be warranted to optimize diagnostic accuracy. Serum AMH levels demonstrate a clear correlation with PCOS severity [59, 92]. The elevated AMH concentrations observed in patients with PCOS primarily reflect the increased numbers of preantral and antral follicles [93]. Mechanistically, AMH elevation may stem from granulosa cell overproduction [94], increased follicular mass [92], or disrupted intrafollicular signaling pathways [95]. In normal physiology, AMH exhibits dynamic fluctuations during the normal menstrual cycle, reflecting its fundamental regulatory roles in folliculogenesis [96]. Its primary functions are to prevent excessive follicular recruitment by inhibiting FSH-induced aromatase activity and to promote dominant follicle selection [97, 98]. However, in PCOS pathophysiology, elevated follicular AMH levels correlate with increased immature oocyte counts, demonstrating its suppressive effect on oocyte maturation [99]. Animal studies further elucidate three roles of AMH: cyclic expression patterns during rat estrus [100], inhibition of oocyte meiosis in rats [101], and follicle development regulation in mice [102]. These findings collectively demonstrate that AMH exerts dual inhibitory effects on folliculogenesis: (1) suppressing primordial follicle recruitment [103, 104], and (2) inducing follicular arrest, thereby increasing antral follicle numbers [104, 105]. Consequently, AMH overproduction in PCOS exacerbates these inhibitory effects, potentially leading to anovulation, as supported by consistent associations between elevated serum AMH levels and ovulatory dysfunction [94, 98, 106].

The levels of INHB in patients with PCOS remain controversial [27–32]. In healthy women, inhibin isoforms exhibit distinct secretory patterns throughout the menstrual cycle. Serum INHB levels peak during the early to midfollicular phase, while INHA levels reach their highest point in the late follicular and luteal phases. These patterns align with in situ hybridization data indicating that the β_A_ subunit is primarily expressed by the corpus luteum and dominant follicle, whereas the β_B_ subunit is localized to the granulosa cells of antral follicles [107]. As proposed by Hayes et al. [25], INHB may serve as a marker for the pool of growing ovarian follicles, whereas INHA reflects the maturity of the dominant follicle and corpus luteum. Existing studies present conflicting findings regarding INHB in PCOS. Some reports indicate elevated INHB levels, possibly due to characteristic luteinizing hormone (LH) hyperstimulation in approximately 50% of PCOS cases, which may enhance granulosa cell production of INHB [48]. Conversely, other studies demonstrate reduced serum and follicular fluid INHB levels in PCOS patients compared to controls [30–32], potentially attributable to insulin resistance (IR). IR may directly impair granulosa cell function in antral follicles and disrupt insulin-like growth factor-Ⅰ (IGF-Ⅰ)-mediated regulation, as evidenced by studies showing IGF-Ⅰ dependency for FSH-stimulated INHB secretion in cultured granulosa cells from small antral follicles [108].

BMI is a well-established clinical factor associated with oligo-anovulation. Obese women exhibit a higher prevalence of oligo-anovulation and generally experience poorer fertility outcomes [109]. Among anovulatory women, Hamilton-Fairly and Taylor [110] observed that PCOS patients typically have higher BMI (> 30 kg/m^2^), while a BMI < 20 kg/ m^2^ is more characteristic of hypogonadotropic hypogonadism. Approximately 50% of PCOS patients are overweight or obese [111], a condition that significantly contributes to the pathogenesis of PCOS by exacerbating both hyperandrogenemia and hyperinsulinemia [112]. These metabolic disturbances are closely interrelated: hyperinsulinemia positively correlates with the degree of hyperandrogenism [113], and both play crucial roles in folliculogenesis disorders [114, 115]. At the follicular level, insulin receptors are expressed on granulosa and theca cells from the primary follicle stage onward [116]. While insulin promotes the transition of primordial to primary follicles, elevated insulin levels in PCOS may disrupt normal folliculogenesis [117]. Similarly, hyperandrogenemia, a hallmark endocrine feature of PCOS, is amplified by increased adipose tissue [118, 119]. Androgens exhibit dual effects on follicular development: they upregulate FSH receptor expression in preantral follicles, stimulating early follicular growth and increasing antral follicle numbers [120], yet simultaneously inhibit FSH and IGF-Ⅰ expression in small antral follicles [121]. This paradoxical regulation aligns with the dynamic expression pattern of androgen receptors, which are abundant in preantral and antral follicles but decline in preovulatory follicles [122].

The mechanisms underlying the differences in AMH levels between obese and non-obese PCOS patients remain unclear. While AMH is typically elevated in PCOS, obese women with PCOS often exhibit lower AMH levels, possibly due to multiple interrelated factors. First, endocrine dysfunction in stromal adipose tissue may impair AMH production [123]. Obesity-related metabolic changes, including altered AMH metabolism, storage, and clearance, could further reduce circulating AMH levels [124]. As reported by Jaswa et al. [35], decreased AMH production by the follicle unit may specifically contribute to this BMI-associated AMH reduction. Additionally, increased leptin production in obesity directly inhibits AMH synthesis via the JAK2/STAT3 pathway [33, 125], while hemodilution effects from increased blood volume may also contribute [126]. Second, LH plays a key role in regulating AMH secretion. Normal-weight women typically have higher LH levels, which stimulate GCs AMH production, potentially explaining their relatively higher AMH levels compared to obese women [59, 127–130]. Obesity, however, is associated with diminished ovarian reserve [131, 132] and follicular dysfunction [61, 133], which may further suppress AMH. Third, obesity-induced hyperandrogenemia (HA) [115] and IR disrupt folliculogenesis. IR and obesity accelerate ovarian aging, leading to early reserve decline [134–137], a phenomenon also observed in non-PCOS women [137–139]. Animal studies suggest that metabolic oxidative stress contributes to gonadal damage, as dietary restriction can restore ovarian reserve [140]. Finally, any factor disrupting the function of the GCs, such as obesity [141, 142]and IR [33, 35, 49, 126, 143–147], likely impairs the production of AMH. This is supported by the higher prevalence of anovulatory cycles in obese women [142]. Notably, metformin treatment in PCOS reduces AMH levels while restoring ovulation, reinforcing the link between IR and AMH dysregulation [148]. Thus, obesity in PCOS may counteract the typical AMH elevation through metabolic, endocrine, and follicular disruptions, ultimately contributing to ovulatory dysfunction.

The mechanisms by which obesity reduces INHB production appear to be independent of PCOS, as an inverse correlation between BMI and serum INHB levels has also been observed in women with normal menstrual cycles [48]. Hyperinsulinemia, which is a hallmark of both obesity and PCOS, likely suppresses INHB production [32, 48, 49, 71], evidenced by the fact that insulin suppression with diazoxide consistently elevates serum INHB levels in overweight PCOS women [32]. Additionally, obesity may directly impair FSH-stimulated inhibin β_B_-subunit production in GCs, further limiting INHB synthesis [48].

The methodological quality of included studies was rigorously assessed using the NOS, with all selected studies achieving scores ≥ 7 points, indicating an overall low risk of bias in this meta-analysis. To investigate potential sources of heterogeneity in the observed association between serum AMH levels and PCOS, we conducted stratified subgroup and meta-regression analyses focusing on obesity status, which identified three major contributing factors: first, significant geographic variations in ethnic differences, dietary patterns, lifestyle behaviors, and socioeconomic status that may systematically influence AMH levels across different populations; second, substantial discrepancies in BMI thresholds for obesity definition across studies may contribute to heterogeneity; and third, variations in participant age ranges (e.g., adolescents vs. reproductive-age women) contribute to heterogeneity, as ovarian reserve function declines with physiological age. This is supported by evidence showing that ovulatory function in PCOS patients may paradoxically improve with advancing age due to follicular pool depletion [149]. While these subgroup analyses helped explain some heterogeneity, significant residual variability persisted, prompting additional sensitivity analyses. These sensitivity tests demonstrated that the pooled effect size for serum AMH levels remained robust and directionally consistent across all model specifications, whereas the analysis of serum INHB levels revealed particularly high heterogeneity (*I²*=90%) that dramatically decreased to just 1% following exclusion of the study by Zhong et al. [68] - an outlier due to its unique use of BMI 24 kg/m² as the obesity cutoff, differing from the ≥ 28 kg/m² threshold employed by other studies. Furthermore, funnel plot asymmetry and statistical tests suggested possible publication bias in the AMH literature, reflecting the known tendency for studies with positive findings to be overrepresented in peer-reviewed publications. These methodological considerations underscore the need for cautious interpretation when applying this meta-analytic findings to clinical practice.

Notwithstanding its findings, this meta-analysis has several limitations that warrant consideration. First, substantial heterogeneity was observed among included studies, primarily stemming from geographical variations in participant recruitment, divergent BMI stratification criteria, and differences in physiological age distributions. This heterogeneity likely reflects both population diversity and methodological variations across studies, as evidenced by funnel plot asymmetry for AMH with several studies falling outside the pseudo 95% confidence limits. Second, this analysis was unable to account for multiple potential confounders reported in original studies, including lifestyle factors (e.g., physical activity, diet, smoking), ethnic variability, inconsistent definitions of obesity (e.g., central vs. general obesity, varying BMI thresholds) and PCOS (e.g., Rotterdam vs. NIH diagnostic criteria), phenotypic heterogeneity (e.g., hyperandrogenism vs. polycystic ovaries), differences in AMH/INHB assay methods, and crucially, unaccounted age effects—all of which may affect the interpretation of the pooled results. Furthermore, the included studies used different assay methods for AMH and INHB (e.g., ELISA, chemiluminescence), which may have contributed to the observed heterogeneity. Subgroup analysis by assay method for AMH was planned but could not be performed because four studies did not report their assay methods (authors unresponsive) and only three studies reported INHB data. Additionally, the mean age of participants in all included studies was below 35 years (range 18–34 years), precluding a subgroup analysis by advanced age (> 35 years). These limitations should be considered when interpreting the results. Third, the limited number of included studies and their predominant case-control designs preclude definitive causal conclusions regarding the temporal relationship between obesity and AMH/INHB levels in PCOS. These limitations collectively underscore the need for future prospective cohort studies that systematically adjust for these confounders and track AMH/INHB trajectories over time in obese PCOS populations. Consequently, while informative, the current findings require cautious clinical interpretation.

## Conclusion

This meta-analysis demonstrates that obese women with PCOS have significantly lower serum AMH and INHB levels compared to non-obese patients with PCOS, revealing distinct impact of obesity on ovarian reserve biomarkers. While AMH maintains diagnostic value in obese PCOS, its obesity-related suppression and the uncertain role of INHB highlight the need for phenotype-specific interpretations, suggesting weight management may be crucial for preserving ovarian function in this population. These findings underscore the importance of considering metabolic status in PCOS biomarker applications while calling for mechanistic studies to elucidate the underlying pathways linking obesity to diminished AMH/INHB levels.

## Supplementary Information

Supplementary Material 1.

Supplementary Material 2.

Supplementary Material 3.

## Data Availability

The data used to support the findings of this study are available from the corresponding author upon request.
