# Supplementary material for: Does obesity affect serum anti-müllerian hormone or inhibin B levels in polycystic ovary syndrome? Results of a systematic review and meta-analysis
Source: Reprod Biol Endocrinol. 2026 Jun 15;24:80. doi: 10.1186/s12958-026-01576-3 (PMC13495322; doi:10.1186/s12958-026-01576-3)
Supplement: Supplementary file 2 [file 12958_2026_1576_MOESM3_ESM.docx]

**Supplemental Table Ⅰ** Search strategy and results for PubMed

| **Search strategy** | | **Results** |
| --- | --- | --- |
| #1 | Polycystic Ovary Syndrome[Mesh] | 20416 |
| #2 | PCOS[Title/Abstract] OR polycystic ovary syndrome[Title/Abstract] | 23456 |
| #3 | #1 OR #2 | 27443 |
| #4 | Non-obese[Title/Abstract] OR nonobese[Title/Abstract] OR normal weight[Title/Abstract] OR normal-weight[Title/Abstract] | 50907 |
| #5 | Anti-Müllerian hormone[Mesh] | 4719 |
| #6 | Anti-Müllerian hormone[Title/Abstract] OR AMH[Title/Abstract] OR Müllerian inhibiting substance[Title/Abstract] OR MIS[Title/Abstract] | 29280 |
| #7 | #5 OR #6 | 29621 |
| #8 | Inhibins[Mesh] | 6372 |
| #9 | Inhibins[Title/Abstract] OR Inhibin-beta subunits[Title/Abstract] OR Inhibin B[Title/Abstract] OR Inhibin-beta[Title/Abstract] OR Inhibin β[Title/Abstract] OR INHB[Title/Abstract] | 3174 |
| #10 | #8 OR #9 | 7666 |
| #11 | #7 OR #10 | 36454 |
| #12 | #3 AND #4 AND #11 | 76 |

**Supplemental Table Ⅱ** Search strategy and results for Cochrane Library

| **Search strategy** | | **Results** |
| --- | --- | --- |
| #1 | MeSH descriptor: [Polycystic Ovary Syndrome] explode all trees | 2182 |
| #2 | (PCOS):ti,ab,kw OR (polycystic ovary syndrome):ti,ab,kw | 5666 |
| #3 | #1 OR #2 | 5666 |
| #4 | (Non-obese):ti,ab,kw OR (nonobese):ti,ab,kw OR (normal weight):ti,ab,kw OR (normal-weight):ti,ab,kw | 24021 |
| #5 | MeSH descriptor: [Anti-Müllerian hormone] explode all trees | 203 |
| #6 | (Anti-Müllerian hormone):ti,ab,kw OR (AMH):ti,ab,kw OR (Müllerian inhibiting substance):ti,ab,kw OR (MIS):ti,ab,kw | 3112 |
| #7 | #5 OR #6 | 3112 |
| #8 | MeSH descriptor: [Inhibins] explode all trees | 124 |
| #9 | (INHB):ti,ab,kw | 54 |
| #10 | (Inhibins):ti,ab,kw OR (Inhibin-beta subunits):ti,ab,kw OR (Inhibin B):ti,ab,kw OR (Inhibin-beta):ti,ab,kw OR (Inhibin β):ti,ab,kw | 381 |
| #11 | #8 OR #9 OR #10 | 409 |
| #12 | #7 OR #11 | 3395 |
| #13 | #3 AND #4 AND #12 | 22 |

**Supplemental Table Ⅲ** Search strategy and results for Embase

| **Search strategy** | | **Results** |
| --- | --- | --- |
| #1 | 'ovary polycystic disease'/exp | 45499 |
| #2 | (pcos OR polycystic) AND ('ovary'/exp OR ovary) AND syndrome:ab,ti | 35996 |
| #3 | #1 OR #2 | 47882 |
| #4 | ('non obese' OR nonobese OR normal) AND ('weight'/exp OR weight) OR 'normal weight':ab,ti | 307945 |
| #5 | 'muellerian inhibiting factor'/exp | 13059 |
| #6 | ('anti müllerian' AND ('hormone'/exp OR hormone) OR amh OR müllerian) AND inhibiting AND substance OR mis:ab,ti | 30855 |
| #7 | #5 OR #6 | 43269 |
| #8 | 'inhibin'/exp | 12271 |
| #9 | ((('inhibins'/exp OR inhibins OR 'inhibin beta') AND subunits OR 'inhibin'/exp OR inhibin) AND b OR 'inhibin beta' OR 'inhibin'/exp OR inhibin) AND β OR inhb:ab,ti | 1917 |
| #10 | #8 OR #9 | 12663 |
| #11 | #7 OR #10 | 54442 |
| #12 | #3 AND #4 AND #11 | 146 |

**Supplemental Table Ⅳ** Search strategy and results for Web of Science

| **Search strategy** | | **Results** |
| --- | --- | --- |
| #1 | TS=(PCOS or polycystic ovary syndrome) | 49575 |
| #2 | TS=(Non-obese or nonobese or normal weight or normal-weight) | 394130 |
| #3 | TS=(Anti-Müllerian hormone OR AMH OR Müllerian inhibiting substance OR MIS OR Inhibins OR Inhibin-beta subunits OR Inhibin B OR Inhibin-beta OR Inhibin β OR INHB ) | 332365 |
| #4 | #1 AND #2 AND #3 | 185 |
