# Supplementary material for: Does obesity affect serum anti-müllerian hormone or inhibin B levels in polycystic ovary syndrome? Results of a systematic review and meta-analysis
Source: Reprod Biol Endocrinol. 2026 Jun 15;24:80. doi: 10.1186/s12958-026-01576-3 (PMC13495322; doi:10.1186/s12958-026-01576-3)
Supplement: Supplementary file 3 [file 12958_2026_1576_MOESM1_ESM.doc]

**MOOSE Checklist**

**Does Obesity Affect Serum Anti-Müllerian Hormone or Inhibin B Levels in Polycystic Ovary Syndrome? Results of a Systematic Review and Meta-analysis**

Mei Jiang,^a#^ Tao Huang,^b#^ Daoyuan Jia,^c^ Ling Huang.^d*^

^a^ Beijing Research Institute of Chinese Medicine, Beijing University of Chinese Medicine, Beijing;

^b^ Department of Nephrology and Endocrinology, Dongzhimen Hospital, Beijing University of Chinese Medicine, Beijing;

^c^ Department of Traditional Chinese Medicine, Hudong Hospital, Shanghai;

^d^ School of Traditional Chinese Medicine, Beijing University of Chinese Medicine, Beijing.

# ^#^ Mei Jiang and Tao Huang contributed equally to this work.

# *Correspondence: Ling Huang, School of Traditional Chinese Medicine, Beijing University of Chinese Medicine, No.11 Beisanhuandong Road, Chaoyang District, 100029, Beijing, P. R. China. E-mail: lhwang@bucm.edu.cn.

| **Criteria** | | **Brief description of how the criteria were handled in the meta-analysis** |
| --- | --- | --- |
| **Reporting of background should include** | |  |
| √ | Problem definition | Anti-Müllerian hormone (AMH) and inhibin B (INHB) are key biomarkers of ovarian reserve and PCOS pathogenesis. However, their differential expression in obese versus non-obese PCOS phenotypes remains unclear. |
| √ | Hypothesis statement | Obese women with PCOS have lower serum AMH and INHB levels compared to non-obese women with PCOS. |
| √ | Description of study outcomes | Serum AMH and INHB levels. |
| √ | Type of exposure or intervention used | Obesity (BMI stratification). |
| √ | Type of study designs used | Included observational studies; excluded conference abstracts and duplicate publications. |
| √ | Study population | Women diagnosed with PCOS (n=15,003 from 38 studies). |
| **Reporting of search strategy should include** | |  |
| √ | Qualifications of searchers | Two independent reviewers (MJ and TH) performed literature screening. |
| √ | Search strategy, including time period included in the synthesis and keywords | Comprehensive search up to September 2025 using keywords: “PCOS,” “AMH,” “INHB,” “obese,” and “non-obese” in Cochrane Library, PubMed, EMBASE, and Web of Science. |
| √ | Databases and registries searched | Cochrane Library, PubMed, EMBASE, Web of Science, PROSPERO (CRD420251168313). |
| √ | Search software used, name and version, including special features | EndNote for deduplication. |
| √ | Use of hand searching | Bibliographies of retrieved articles were reviewed. |
| √ | List of citations located and those excluded, including justifications | PRISMA flow diagram details screening process (304 identified, 38 included). |
| √ | Method of addressing articles published in languages other than English | No language restrictions applied. |
| √ | Method of handling abstracts and unpublished studies | Contacted authors for missing data. |
| √ | Description of any contact with authors | Corresponding authors were contacted for unreported data (e.g., SDs). |
| **Reporting of methods should include** | |  |
| √ | Description of relevance or appropriateness of studies assembled for assessing the hypothesis to be tested | Included studies compared AMH/INHB levels in obese vs. non-obese PCOS women. |
| √ | Rationale for the selection and coding of data | Extracted mean ± SD for AMH/INHB, with imputation for missing SDs. |
| √ | Assessment of confounding | Subgroup analyses addressed geographic, BMI, and age variability. |
| √ | Assessment of study quality, including blinding of quality assessors; stratification or regression on possible predictors of study results | NOS scores ≥7 for all studies (low risk of bias). |
| √ | Assessment of heterogeneity | Evaluated using *I²* and Cochran’s Q tests; random-effects models for *I²* ≥ 50%. |
| √ | Description of statistical methods in sufficient detail to be replicated | SMDs with 95% CIs; subgroup/sensitivity analyses; RevMan 5.3. |
| √ | Provision of appropriate tables and graphics | PRISMA flowchart, forest plots, subgroup tables, funnel plot (AMH only). |
| **Reporting of results should include** | |  |
| √ | Graph summarizing individual study estimates and overall estimate | Forest plots for AMH (Figure 2A) and INHB (Figure 2B). |
| √ | Table giving descriptive information for each study included | Table 1 summarizes study details (location, BMI, age, etc.). |
| √ | Results of sensitivity testing | AMH results robust; INHB results dependent on one study. |
| √ | Indication of statistical uncertainty of findings | Reported 95% CIs, *I²* values, and *P*-values. |
| **Reporting of discussion should include** | |  |
| √ | Quantitative assessment of bias | Funnel plot asymmetry suggested potential AMH publication bias. |
| √ | Justification for exclusion | Detailed in PRISMA flowchart (e.g., non-extractable data). |
| √ | Assessment of quality of included studies | All studies scored ≥7 on NOS (Table 2). |
| **Reporting of conclusions should include** | |  |
| √ | Consideration of alternative explanations for observed results | Discussed metabolic confounders (e.g., insulin resistance). |
| √ | Generalization of the conclusions | Obesity may suppress AMH/INHB; weight-stratified interpretation needed. |
| √ | Guidelines for future research | Investigate obesity-AMH/INHB pathways. |
| √ | Disclosure of funding source | This work was supported by the Fundamental Research Funds for the Central Universities (No. 2025-JYB-KYPT-04) and the Beijing University of Chinese Medicine vertical research and development fund (No. 2023-ZXFZJJ-058). |
